# Short term micronutrient-antioxidant supplementation has no impact on a serological marker of gastric atrophy in Zambian adults: retrospective analysis of a randomised controlled trial

**DOI:** 10.1186/1471-230X-14-52

**Published:** 2014-03-25

**Authors:** Violet Kayamba, Mumba Chomba, Paul Kelly

**Affiliations:** 1 Department of Internal Medicine, Tropical Gastroenterology & Nutrition group, University of Zambia School of Medicine, Nationalist Road, Lusaka, Zambia; 2 Blizard Institute, Barts & The London School of Medicine and Dentistry, Turner Street, London, UK

## Abstract

**Background:**

Gastric cancer is a major contributor to cancer deaths in Zambia but, as elsewhere, no preventive strategies have been identified. We set out to investigate the possibility of reducing gastric atrophy, a premalignant lesion, using micronutrient-antioxidant supplementation.

**Methods:**

We analysed 215 archival samples from a randomised controlled trial of micronutrient-antioxidant supplementation carried out from 2003 to 2006. Participants were randomised to receive either the supplement or placebo and had been taking the allocated intervention for a mean of 18 (range 14–27) months when the samples used in this study were taken. We used low pepsinogen 1 to 2 (PEP1:2) ratio as a surrogate marker of gastric atrophy. A PEP 1:2 ratio of less than three was considered low. HIV serology, age, nutritional status, smoking, alcohol intake and gastric pH were also analysed. Ethical approval was obtained from the University of Zambia Biomedical Research Ethics Committee (011-04-12). The randomized trial was registered (ISRCTN31173864).

**Results:**

The overall prevalence of low PEP 1:2 ratio was 15/215 (7%) and it did not differ between the placebo (8/103, 7.8%) and micronutrient groups (7/112, 6.3%; HR 1.24; 95% CI 0.47-3.3; *P* = 0.79). The presence of low PEP 1:2 ratio was not influenced by HIV infection (HR 1.07; 95% CI 0.37-3.2; *P* =0.89) or nutritional status but it inversely correlated with gastric pH (Spearman’s rho = -0.34; *P* = 0.0001). Age above 40 years was associated with atrophy, but neither alcohol nor smoking had any influence.

**Conclusion:**

Short term micronutrient supplementation does not have any impact on PEP 1:2 ratio, a serological marker of gastric atrophy. PEP 1:2 ratio inversely correlates with gastric pH.

## Background

Gastric cancer is a major contributor to cancer deaths in Zambia but, as elsewhere, no appreciable preventive strategies have been identified. Low intake of fruits and vegetables has been associated with increased risk of gastric adenocarcinoma in the United States of America [1]. This appears to be also true for patients in Zambia, as we reported in a case control study that low fruit intake in the diet was associated with gastric cancer [2]. Furthermore, isoprostane excretion was increased in cancer cases, suggesting that the association with low fruit intake might be mediated via antioxidant status. While cancer is associated with poor antioxidant status, it is less well established if the same applies to premalignant lesions.

The sequence of gastric premalignant lesions was first defined by Correa in 1975 [3]. Premalignant lesions include atrophy, intestinal metaplasia and dysplasia [4]. These are believed to be present for several years before undergoing malignant transformation. The best prospect for prevention of cancer would be to halt the progress or reverse these lesions, using an intervention which addresses some fundamental aspect of carcinogenesis. Repeated endoscopy would be a good way to evaluate presence or reversal of premalignant lesions, but it is invasive and not without sampling error. Low pepsinogen 1 to 2 ratio (PEP 1:2) has been shown to correlate very well with gastric atrophy [5-8] and is thus an attractive alternative for determining the presence of gastric atrophy. We have previously shown that the prevalence of serologically diagnosed gastric atrophy among patients with normal upper gastrointestinal endoscopies was as high as 28% (26/94) with 23% (6/26) of these being less than 45 years old [9].

There is conflicting evidence regarding the benefit of micronutrient supplementation in premalignant gastric lesions. Most studies have failed to sufficiently prove any beneficial effect of this supplementation on the progression to gastric cancer [10-12]. Vitamin C has no effect on the reduction of infections in patients with gastric atrophy [13]. However, other studies have shown that there is significant regression of gastric atrophy after anti-oxidant supplementation [1,14]. The findings in the large trial in Linxian, China, showed that giving beta-carotene, vitamin E and selenium supplements for 5 years had an effect on the reduction of gastric cancer risk [15]. A study done in mice showed that vitamin C supplementation does not protect against *Helicobacter pylori (H. pylori)* gastritis or gastric premalignancy [16]. Beno *et al.*[17] reported that the serum levels of vitamins A, C and E, selenium, zinc and copper were low in patients with premalignant gastric lesions in Slovakia.

These studies mainly investigated the effects of vitamins C, E, A and selenium, and did not include other micronutrients. These studies also used histological changes to assess gastric atrophy. We set out to test the hypothesis that micronutrient and antioxidant supplementation could lead to the regression of gastric atrophy determined by low PEP 1:2 ratios in a Zambian population.

## Methods

This was a retrospective analysis of samples from a randomised, placebo-controlled trial of multiple micronutrient and antioxidant supplementation which was conducted in Misisi Township in Lusaka, Zambia between 2003 and 2006 [18]. Misisi is one of the poorest and most densely populated townships in Lusaka, with a prevalence of *H. pylori* infection of 81% [19]. For this study, we used samples collected in 2005 from volunteers who had been in the trial for not less than 12 months. As the allocation was random, it was assumed that at the start of the study the proportion of subjects with gastric atrophy would be the same in both groups, and thus any difference noted at the end of the study would be attributable to the supplementation. Ethical approval was obtained from the University of Zambia Biomedical Research Ethics Committee, ref 011-04-12. The randomized trial was registered as ISRCTN31173864.

### Conduct of the trial during which samples were collected

During the controlled trial, all consenting residents above the age of 18 years, living in the designated area were eligible to participate in the study. There were no exclusion criteria. In total, 500 residents volunteered to participate in the study, half of whom were randomly allocated to receiving either micronutrient supplementation or placebo. The multiple micronutrient and the placebo tablets were both prepared and supplied by Dnask Farmaceutic Industri (Ballerup, Denmark). The supplement tablets contained multiple micronutrients at around 1.5-2 times Recommended Nutrient Intakes (Table 1). Details of the randomization have been previously reported [18]. Blood samples were collected from these volunteers at the start of the study and annually until the end of the study, and stored at -80°C in a secure laboratory. Compliance was measured by counting unused pills and was estimated at 95% [18]. Nutritional assessment at baseline included height, weight (to determine the body mass index), and mid upper arm circumference; fat mass and lean mass were measured by impedance (Body Stat 1500, Douglas, Isle of Man, UK). Gastric pH, measured in fasting participants by endoscopic aspiration of gastric juice, was measured as previously described [20].

**Table 1:** Composition of the micronutrient tablet

**Micronutrient**	**Amount**	**Reference Nutrient Intake (RNI)**
Beta-Carotene	4.8 mg	4.2 mg equivalent
Ascorbic acid (vitamin C)	70 mg	40 mg
Cholecalciferol (vitamin D_3_)	5 μg	_
Tocopherol (vitamin E)	10 mg	4 mg (uncertain)
Thiamine (vitamin B_1_)	1.4 mg	1.0 mg
Riboflavin (vitamin B_2_)	1.4 mg	1.3 mg
Niacin	18 mg	17 mg
Pyridoxine (vitamin B_6_)	1.9 mg	1.4 mg
Cyanocobalamin (vitamin B_12_)	2.6 μg	1.5 μg
Folic acid	400 μg	200 μg
Iron	30 mg	14.8 mg (women), 8.7 mg (men)
Zinc	15 mg	9.5 mg
Copper	2 mg	1.2 mg
Selenium	65 μg	75 μg
Iodine	150 μg	140 μg

RNI from reference [21].

### Serology

To determine the presence of severe gastric atrophy, PEP 1:2 ratios were determined using ELISA kits for pepsinogen 1 and 2 (Biohit, Helsinski, Finland) and the manufacturer’s instructions were followed. A ratio less than 3.0 was used to signify the presence of severe gastric atrophy. VK and MC were completely blinded to the allocations while running the samples. The coding of these samples was only broken by PK after completion of data entry.

### Data analysis

Statistical analysis was carried out using STATA 12.1 (STATA Corp. College Station. TX). For continuous variables showing Gaussian distribution, the Kruskal-Wallis test was used to compare the two groups. For categorical variables, Fisher’s exact test was used. Backwards step wise unconditional logistic regression was used to assess the contributions of different factors to gastric atrophy.

## Results

**Figure 1:**
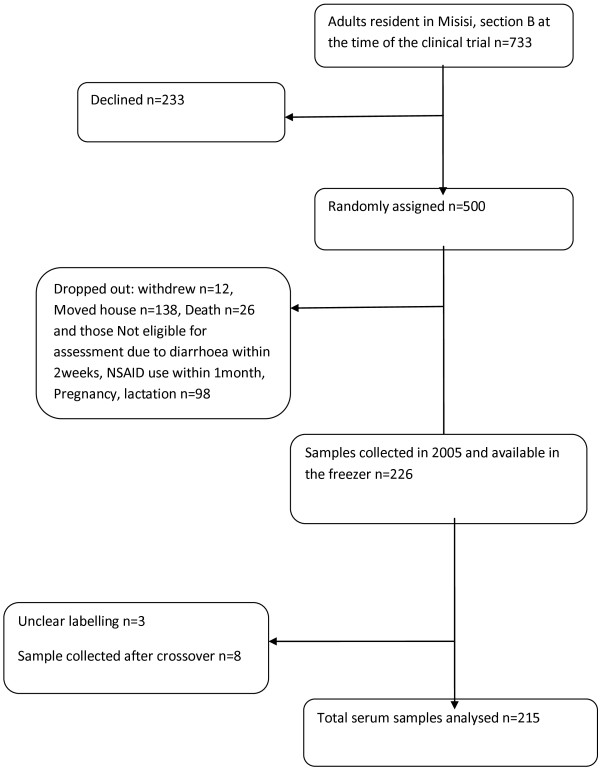
Flow charts showing how the final samples analysed were obtained.

A total of 226 serum samples collected in 2005 were available for analysis. Eleven samples were, however, left out of the analysis as three were not clearly labelled and eight were collected after crossover from micronutrient to placebo in the original trial [18], leaving a total of 215 samples. The flow of collected samples used in this study was as outlined in Figure 1. Subjects from whom these samples were collected had therefore received either micronutrient supplementation for a median of 18 months (IQR 16–19) or placebo for 19 months (IQR 18–20). Demographic characteristics and potential confounders are shown in Table 2.

**Table 2:** Demographic characteristics of the participants

	**Supplementation group (n = 103)**	**Placebo group (n =112)**	***P***
**Mean age in years**	37+/-13	37.1+/-13	0.869
**Number of females n (%)**	70 (68.0)	71 (63.4)	0.566
**Education n (%)**			
** Primary**	73 (70.9)	88 (78.5)	
** Secondary or better**	30 (29.1)	24 (21.4)	0.333
**Mean Body Mass Index (kg/m**^**2**^**)**	22.6+/-3.8	22.8+/-5.9	0.581
**HIV positive n = 66 (%)**	32 (48.4)	34 (51.5)	0.592
**Mean Middle Upper Arm Circumference (MUAC)**	27.3+/-3.3	27.4+/-4.2	0.705
**Mean Body fat (kg)**	29.9+/-9.4	29.5+/-9.8	0.547
**Mean Lean mass (kg)**	70.0+/-9.3	69.9+/-11.0	0.630
**Median (IQR) follow up (months)**	18 (16–19)	19 (18–20)	0.0001
**pH n = 121**	3.3+/-2.2	3.2+/-2.2	0.881
**Tobacco use n (%)**	15 (14.5)	23 (20.5)	0.286
**Cannabis n (%)**	4 (3.8)	2 (1.8)	0.430
**Alcohol n (%)**			18 (16.1)
** Everyday**	12 (11.6)	18 (16.1)	
** Not every day**	39 (37.9)	33 (29.5)	0.661

### Gastric atrophy (low PEP 1:2 ratio)

The overall prevalence of gastric atrophy in these healthy volunteers was 15/215 (7%). There was no significant difference in the prevalence of low PEP 1:2 ratios between the participants on micronutrient supplementation, 8/103(7.8%) and those on placebo, 7/112 (6.3%) Hazard Ratio (HR) 1.24; 95% CI 0.47-3.3; *P* = 0.79. Analysis of the PEP 1:2 ratios of all the samples as continuous variables did not yield any statistically significant difference (*P* = 0.32). The mean ratio among the supplementation group was 5.8 (IQR 4.3-6.9), while that among the placebo group was 5.9 (IQR 4.5-8.0).

### HIV infection

Of the serum samples analysed, 180 had HIV tests performed in the previous trial, and 66 (37%) were positive. Of these subjects with HIV infection, 5/66 (7.6%) had gastric atrophy, which did not differ significantly from HIV negative subjects among whom 8/114 (7.0%) had atrophy (HR 1.07; 95% CI 0.37-3.2; *P* =0.89).

### Gastric pH

Measurements of pH in gastric aspirates taken while fasting were available on 121 participants. Gastric pH and low PEP 1:2 ratios were inversely correlated (Spearman’s rho = -0.34; *P* = 0.0001). Of the 60 participants randomised to placebo for whom pH measurements were available, 19 (32%) had a fasting gastric pH of more than 4 (which was taken as significant hypochlorhydria) compared to 26 of 61 (43%) participants allocated to micronutrient-antioxidant supplementation (HR 1.35, CI 0.84-2.16, *P* = 0.26).

### Nutritional status

Parameters of nutritional status analysed in the main trial were considered in this analysis. There was no correlation between gastric atrophy and nutritional status (Table 3).

**Table 3:** Correlation with pepsinogen 1:2 ratio

	**Spearman’s** ***ρ***	***P*** **value**
**pH (n = 121)**	-0.345	0.0001
**BMI**	-0.003	0.967
**MUAC**	0.005	0.947
**Body fat**	-0.107	0.122
**Lean mass**	0.075	0.280
**Impedance**	-0.043	0.542

### Multivariate analysis

A multivariate logistic regression was carried out including several factors to try and ascertain any relation to severe gastric atrophy (Table 4). Alcohol and smoking did not show any influence on gastric atrophy. The amount of alcohol being consumed was also taken into consideration, and categorised as those taking every day and not every day. Even after taking this into consideration, there was still no significant difference between the atrophy subjects and the ones without atrophy. The only influence found to be significant was age, as subjects above the age of 40 years were found to be more at risk of having gastric atrophy.

**Table 4:** Logistic regression analysis of atrophy (measured as low pepsinogen 1:2 ratio) in relation to potential determinants

	**Odds ratio**	**95% CI**	***P***
Sex (female)	0.59	0.21-1.66	0.32
Age (Over 40 years)	0.28	0.09-0.83	0.02
Micronutrient-antioxidant supplementation	1.06	0.38-3.00	0.91
HIV positive	1.51	0.44-5.20	0.52
Low education	1.62	0.41-6.43	0.49
Alcohol use	0.85	0.58-1.24	0.40
Tobacco use	0.86	0.17-4.30	0.86

## Discussion

Gastric cancer remains a major cause of cancer mortality in Zambia [22]. We set out to investigate the possibility that micronutrient supplementation could reduce the occurrence of low PEP 1:2 ratio, which is a surrogate marker of gastric atrophy. We found that an average of 18 months of micronutrient supplementation made no impact on its occurrence among healthy adult Zambians. HIV infection was also found not to have any influence on atrophy. Gastric atrophy was more likely among participants above the age of 40 years, while there was an inverse correlation with fasting pH, which is consistent with our previous findings [20]. Alcohol and smoking also did not show any influence on gastric atrophy.

The study participants were randomly allocated to either supplementation or placebo in the main trial. We made a reasonable assumption at since the participants were randomly allocated at enrolment then the prevalence of atrophy was not significantly different in the two groups. It is well understood that any differences in groups that have been randomly allocated are merely due to chance [23]. This is a premise on which randomised controlled trials are based. There was essentially no difference in the basic demographics of the two groups of participants used in this analysis. However, on average participants allocated to the placebo group were followed up for one month longer than the supplementation group. This was statistically significant (*P* = 0.0001). If the supplementation had any effect on development of atrophy, this difference would still have been seen despite the longer follow-up in the placebo group. It seems unlikely that this difference in duration of follow-up (which is determined by the date the sample was collected) could explain a lack of apparent benefit. On the other hand, 18 months might be too short to demonstrate the effect of these supplementations and it is possible that a longer follow-up (5–10 years) might yield different results.

Gastroscopies were done on some of the participants in the main trial to determine the fasting gastric pH. Unfortunately, no gastric biopsies were obtained as this was not part of that study protocol. We therefore, did not have any data on the histological diagnosis of gastric atrophy or the degree of atrophic changes. Analysis of PEP 1:2 ratios as continuous variables did not yield any statistically significant difference between the two groups.

We know from our previous work that the presence of *H. pylori* antibodies is very common in this population [19]. *H. pylori* infection has an influence on the presence and persistence of gastric atrophy but it was not tested in the main trial and we did not have the appropriate samples to test for active infection. Therefore, the presence of *H. pylori* infection was not checked in this retrospective analysis of stored serum samples. This could not have affected the results as none of the patients received treatment to eradicate the infection during the study follow-up.

The adherence (pill count) of the subjects was at more than 95%, as reported in the main trial [18]. Participants were drawn from an impoverished community in Zambia where micronutrient deficiency is common. If supplementation had an effect, then it would be more obvious in such a population than in a generally well nourished community. Our study was not designed to assess the nutritional impact on intestinal metaplasia, which is another step in the carcinogenetic pathway.

The population prevalence of gastric atrophy in Zambia is unknown, but in a hospital based case–control study [9], we found that up to 28% of healthy controls had serological evidence of severe gastric atrophy. In this study, we used serum samples from healthy community volunteers and found that the prevalence of atrophy was much less at 7%. However, in the current study the mean age was 37 years whereas it was 55 years in the case–control study. We have demonstrated that gastric atrophy tends to be more common with advancing age, which could explain the different prevalence. On the other hand, these findings might reflect a higher frequency of incipient gastroduodenal pathology in hospital based controls.

HIV positive patients have in the past been reported to have higher gastric pH than their HIV negative counterparts [20]. Neither the aetiology nor the consequence of this finding is clear. In addition, HIV infection was not found to be associated with gastric cancer [9]. In this study, we found no correlation between HIV infection and gastric atrophy, suggesting that the hypochlorhydria seen in HIV infection cannot be explained by the loss of normal gastric mucosa cells. More work still needs to be done in order to find out why HIV infected patients have high pH and the consequence of this as there seem to be no connection with gastric cancer or it precursor lesions [9].

We were also interested to see if alcohol and smoking had any influence on gastric atrophy. It remains unclear at which exact stage (if at all) of the carcinogenic pathway alcohol and smoking influence gastric carcinogenesis. We found that these two factors have no influence on gastric atrophy.

There is a shortage of data on gastrointestinal malignancies in Africa, and further work is needed to determine if there might be any scope for nutritional interventions to reduce risk.

## Conclusions

An average of 18 months of micronutrient-antioxidant supplementation has no impact on the occurrence of low PEP 1:2 ratio, which is a surrogate marker of gastric atrophy. The presence of HIV infection has no influence on atrophy. Low PEP 1:2 ratios were more likely among participants above the age of 40 years, while there was an inverse correlation with fasting pH. Alcohol and smoking also did not show any influence on gastric atrophy.

## Competing interests

The authors declare that they have no competing interest.

## Authors’ contributions

VK and PK designed the study; VK collected and sorted the serum samples; VK and MC did the Enzyme-linked Immunoabsorbant Assays on the samples; PK performed the statistical analysis; VK and PK analysed the data; VK, MC and PK contributed to the write-up of the manuscript. All authors read and approved the final manuscript.

## Pre-publication history

The pre-publication history for this paper can be accessed here:

http://www.biomedcentral.com/1471-230X/14/52/prepub
